# Significant Association between the T2 Values of Vertebral Cartilage Endplates and Pfirrmann Grading

**DOI:** 10.1111/os.12727

**Published:** 2020-06-24

**Authors:** Yi Cao, Qing‐wei Guo, Ye‐da Wan

**Affiliations:** ^1^ Department of Radiology Tianjin Hospital Tianjin China; ^2^ Department of Radiology First Teaching Hospital of Tianjin University of Traditional Chinese Medicine Tianjin China

**Keywords:** Cartilage disease, Intervertebral disc degeneration, Intervertebral discs

## Abstract

**Objective:**

The T2 value of lumbar cartilage endplates was measured using the T2 mapping imaging technique, aiming to explore the correlation between the T2 value and Pfirrmann grading of intervertebral discs.

**Methods:**

A total of 130 patients with lumbar spine MR examination due to persistent low back pain were enrolled, including 71 men and 59 women (age: 21–63 years). Lumbar Modic changes and Schmorl nodules were recognized by conventional T1WI and T2WI images in 49 patients, and these patients were excluded from the study. A total of 81 patients were enrolled in this study, including 45 men (45.16 ± 12.20 years) and 36 women (43.33 ± 11.27 years). Pfirrmann (Pm) grading of each lumbar disc was performed based on conventional T2WI median sagittal images and the position of cartilage endplates (CEP) was determined by IDEAL‐SPGR images. Meanwhile, the T2 mapping technique was used to obtain T2 values of cartilage endplates. The T2 values of CEP corresponding to different Pm grade discs were compared, and the correlation between the T2 value and the Pm grade of intervertebral discs was analyzed.

**Results:**

The T2 values of cephalic and caudal CEP of L_1–2_ in Pm grades I–II, Pm grades III, and Pm grades IV–V were 61.96 ± 5.89 ms, 54.45 ± 3.29 ms, 42.47 ± 3.69 ms and 64.35 ± 5.93 ms, 55.28 ± 3.97 ms, 44.75 ± 2.12 ms, respectively. For cephalic and caudal CEP of L_2–3_, the T2 values in Pm grades I–II, Pm grades III, and Pm grades IV–V were 62.96 ± 6.93 ms, 55.19 ± 4.02 ms, 48.67 ± 4.56 ms and 65.51 ± 6.49 ms, 57.16 ± 5.55 ms, 52.05 ± 4.20 ms, respectively. The T2 values of cephalic and caudal CEP from L_3–4_ to L_5_–S_1_ in Pm grades I–II, Pm grades III, and Pm grades IV–V were (63.72 ± 5.76 ms, 53.96 ± 6.52 ms, 48.05 ± 5.00 ms), (65.46 ± 6.37 ms, 55.70 ± 7.50 ms, 48.10 ± 3.27 ms); (66.34 ± 7.68 ms, 56.76 ± 9.48 ms, 47.80 ± 4.33 ms), (64.44 ± 4.65 ms, 59.30 ± 8.80 ms, 47.30 ± 5.78 ms), (65.32 ± 5.11 ms, 55.33 ± 6.65 ms, 48.18 ± 5.37 ms), and (63.47 ± 4.92 ms, 50.32 ± 8.86 ms, 44.77 ± 4.69 ms), respectively. There were significant differences in T2 values of cartilage endplates between the Pm grades I–II, III, and IV–V of intervertebral discs (*P* = 0.000). T2 values corresponding to Pm I–II grades were higher than those in Pm III grade, while T2 values in Pm grades IV–V were lowest. The T2 value of the L_4–5_, L_5_–S_1_ segment endplates was highly correlated with the Pm grades (*r* = −0.711, −0.721, −0.796, −0.745; *P* = 0.000) and that of L_1–2_, L_2–3_ endplates were moderately correlated (*r* = −0.542, −0.562, −0.637, −0.612; *P* = 0.000).

**Conclusion:**

The T2 values of cartilage endplates revealed varying degrees of degeneration of intervertebral discs, and more severe degeneration corresponded to lower T2 values. Measurement of changes in the T2 value through cartilage endplates can be useful for the diagnosis of early intervertebral disc degeneration and the prevention of disc degeneration.

## Introduction

Low back pain and osteoarthritis contribute to disability worldwide and the consequent impacts, including absence at work and on healthcare, have a serious socioeconomic burden^1^, ^2^, ^3^. Intervertebral disc degeneration disease (IDDD) is a common reason for these two diseases^4^. The vertebral endplates have a critical role in the prevention and early diagnosis of IDDD; therefore, it is significant to shed light on the pathogenesis of the degeneration of vertebral endplates.

The cartilage endplate (CEP) is one of the main components of the intervertebral disc, combining the annulus fibrosus and the nucleus pulposus and ensuring the structural and mechanical integrity of the organ. The vertebral endplates are the strongest part of the disc, located at the top and bottom of each intervertebral disc. They transport the cells and other required nutrients in and out of the disc, to keep the disc alive and to prevent degeneration^5^, ^6^. Degeneration of intervertebral discs is mainly due to the degeneration of CEP, which obstructs the nutrition supply to the disc^7^.

Using a noninvasive imaging technique for evaluating CEP is necessary because of its clinical correlation with disc degeneration^8^. Disc degeneration is often measured with the semiquantitative Pfirrmann grading method using T2‐weighted images with five grades based on morphology and signal intensity^9^. Prior to structural changes in vertebral endplates with degeneration, the cartilage tissue is subjected to biochemical alterations, including a loss of proteoglycans and a concomitant decrease of hydration^10^, ^11^. Hence, the diagnosis of early CEP degeneration requires a high‐resolution technique for morphological evaluation and physiologic techniques to detect biochemical abnormalities.

The CEP is an extremely thin and delicate anatomical structure, and most research studies focus on cadaver specimen measurement^12^, ^13^. To be clinically practical, the imaging identification of CEP needs to satisfy high resolution, high signal‐to‐noise ratio (SNR), and a short time of echo (ET) with optimal sequence parameter^14^. Conventional MRI using T2‐weighted imaging takes a long time when multiple images are required, is not sensitive to early morphological changes, and provides no predictive quantitative biomarker profile of early degeneration^15^, ^16^. IDEAL (iterative decomposition of water and fat with echo asymmetry and least‐squares estimation) with highly SNR‐efficient and fat‐saturated SPGR (spoiled gradient echo), has been considered the clinical reference standard for morphologic quantification and volumetric assessment of cartilage because of its high resolution^14^, ^17^, ^18^.

A previous study performed SPGR imaging on lumbar CEP of cadavers, and the CEP of all lumbar spine specimens were clearly displayed by the SPGR sequence. Importantly, the CEP on the MR images had morphological abnormalities, including thinner height, irregular, erosion, and defects, which were confirmed by anatomical examination^19^. Hence, the IDEAL‐SPGR sequence can provide steady water‐fat separation with high SNR and resolution with significantly shorter scanning time.

This study applies the IDEAL‐SPGR sequence to investigate the lumbar cartilaginous endplate *in vivo*, which can accurately display the morphology and location of endplates. In addition, T2 mapping is sensitive to changes in collagen (content/alignment) and water, and it can quantitatively reveal the variation of biochemical material^20^. T2 values (i.e. voxel‐specific T2‐relaxation times) are determined from a series of continually increasing echo times by evaluating the inherent property of the macromolecular environment, including water, protein, collagen, fat, and other solutes. To be more specifically, T2 values correlate to the concentration of water molecules and their interaction with collagenous fibers (amount and orientation), in which the dipole interaction caused by anisotropic motion of water molecules plays a key role^21^. T2 values have been shown to inversely correlate with degeneration grade and were first used to assess the biochemical changes in early degeneration of articular cartilage^20^. T2 values also reported to have the capacity to discriminate the nucleus pulposus from the annulus fibrosus^22^. T2 mapping technology has the characteristics of non‐invasiveness and reproducibility. It can be used to quantitatively analyze the changes in the articular cartilage and intervertebral disc matrix composition by measuring the T2 relaxation time. Watanabe *et al*. describe the potential benefits of standard axial T2 mapping in the lumbar spine and suggest that T2 mapping is a reliable and valid method in biochemical cartilage imaging^23^.

Therefore, this study aims to investigate the correlation between Pfirrmann grading and T2 values, where IDEAL‐SPGR provides the precise position for T2 component measurement. Specifically, this research addresses three main points as follows. First, we analyze the difference in T2 values between adjacent cephalic and caudal CEP of the intervertebral disc in different Pm grades. Second, we explore the relationship of the T2 value and the Pm grade through correlation analysis. Finally, based on the results of the above, using T2 mapping technology, we attempt to provide a quantitative evaluation method for early degeneration of lumbar cartilage endplates using T2 mapping technology.

## Patients and Methods

All 130 patients (age: 21–63 [44.13 ± 11.26] years; 71 men, 59 women) included in this study signed informed consent forms. These patients underwent functional MR regular examination for lumbar vertebra in Tianjin Hospital from June to December in 2016.

Inclusion criteria were as follows. First, clinical symptoms were sustained or the patient had recurrent chronic low back pain, with or without lower extremity radiation pain. Second, patients could cooperate to complete the magnetic resonance examination and measure the T2 value of the lumbar cartilage endplate using the T2 mapping technique. Third, according to the semi‐quantitative visual grading system proposed by Pfirrmann in 2001^9^, intervertebral discs from L_1_ to S_1_ were graded using the T2WI median sagittal images. Takashima *et al*.^24^ report there is no significant difference in the T2 values of the nucleus pulposus between Pfirrmann (Pm) IV and PmV, and it is difficult to distinguish between PmIV and PmV. Here, we divided patients into three groups, G1: Pm I–II (for apparently healthy discs of children and young adults); G2: Pm III (for discs with reduced T2 signal intensity); and G3: Pm IV–V (for discs with progressively decreased height and T2 signal)^9^, ^25^. Fourth, correlation analysis was performed to explore the relationship between T2 values and Pm grades. Finally, the study design used the prospective research method.

Exclusion criteria: long‐term heavy physical work; BMI ≥28 kg/m^2^; having a history of spinal trauma, surgery, congenital malformations of the spine, and various diseases involving the spine, such as inflammation, tumors, blood system diseases, and metabolic bone diseases; claustrophobia, metal implants, or other MR contraindications; and scoliosis >15°.

### *Image Acquisition*

All imaging was performed with the approval of the Regional Committee for Ethics of Tianjin Hospital. MRI was conducted using a Discovery MR 750W 3T scanner (GE Healthcare, Waukesha, WI) employing a CTL surface coil with full coverage of the neck, chest, and waist. Subjects lay in a supine position on the CTL coil, and the positioning line was set at the level of the third lumbar vertebra. Scanning sequences included regular T1WI, T2WI, IDEAL‐SPGR, and T2 mapping and imaging was performed in the sagittal plane for all sequences.

Scanning parameters of T2WI sequence (FRFSE sequence) were: time of repetition (TR) was 4000 ms, time of echo (TE) was 121 ms, number of excitations (NEX) was 2, slice thickness was 4 mm with the slice gap (SP) of 0.5 mm, 10 slices, 320 × 320 mm field of view (FOV), 352 × 320 matrix size, and the scanning time (ST) was 2 min 16 s. T1WI sequence (i.e. FLAIR sequence) used a TR of 2950 ms, TE of 22 ms, TI of 1050 ms, a 142° flip angle, 320 × 320 mm^2^ FOV, 320 × 224 voxel matrix size, 1.5 mm slice thickness with 0.5 mm SP, 10 slices, 2 NEX, and an ST of 2 min 55 s.

Parameters for IDEAL–SPGR imaging included a TR of 6.7 ms, TE of 3.0 ms, a 320 × 224 matrix, 300 × 300 mm^2^ FOV, 3 mm slice thickness with 0 mm SP, 12 slices, 3 NEX, a 15° flip angle, and an ST of 4 min 30 s. T2 mapping used the multi‐echo SE sequence, including 8 echo collections in total, a TR of 1000 ms and 8 TE values (8.5, 16.9, 25.4, 33.9, 42.3, 50.8, 59.2, 67.7 ms), 3 mm slice thickness with 1 mm SP, 10 slices, 2 NEX, 300 × 300 mm^2^ FOV, 256 × 192 matrix size, and an ST of 6 min 26 s.

### *Image Data Measurement and Processing*

Images were interpreted independently by two MRI diagnosticians of different levels using the double‐blind method. T1WI, T2WI images showing the existence of lumbar Modic changes or Schmorl nodules were excluded in this study. The Pm grading was determined by two diagnosticians based on T2WI median sagittal images; when the opinions were inconsistent, the grade was based on the assessment of the senior medical diagnostician.

The nutrition diffusion between intervertebral discs is largely dependent on the central section of the CEP. From the perspective of biomechanics, the reassignment from the loading of the intervertebral disc and the stress conduction also mainly relies on CEP and most of the stress acts on the weakest area, the central region^26^, ^27^, ^28^. Therefore, the Region of Interest (ROI) was set at the center of the CEP in this study, which shows the most prominent and representative characteristics of degeneration of intervertebral discs.

**Figure 1:**
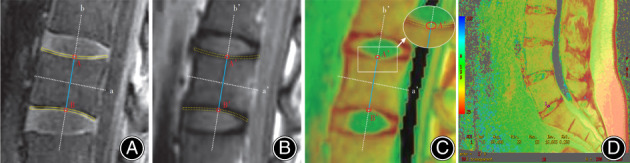
Schematic diagram of the ROI setting for cartilage endplates (CEP). (A) CEP shows high signal intensity on the median sagittal image of IDEAL‐SPGR, and the midpoint of cephalic and caudal CEP is marked with A and B, respectively; (B) the ROI on the T2 original images is located according to the IDEAL‐SPGR image. (C) T2 mapping pseudo‐color map. (D) Illustration of the practical ROI measurement.

IDEAL‐SPGR images are capable of displaying the profile of CEP clearly, based on which the ROI was drawn on IDEAL‐SPGR images. Then, the ROI was co‐registered on the corresponding T2 mapping slice to calculate the T2 values. First, the midpoint of the anterior and posterior margins of each vertebral body was connected on the maximum amplification of the IDEAL‐SPGR median sagittal image to make a line, named “a” (Fig. 1A). Line “b” was perpendicular to line “a,” based on which the distance between the upper and lower CEP was recorded as AB, shown in Fig. 1A. The lower and upper CEP of each intervertebral disc were defined as the cephalic endplate and the caudal endplate, respectively.

In the post‐processing workstation, the T2 map module was selected using the FuncTool software and the confidence interval was set to 95% on both sides with a color threshold of 20 ms. The color scale which controls the T2 values in the range of 25–75 ms was adjusted, and the T2 mapping pseudo‐color map that was obtained was automatically corrected. Similar to the processing of IDEAL‐SPGR images, a′ was a line connecting the midpoints of the anterior and posterior margins of each vertebral body, while b′ was the midperpendicular of a′. We overlapped line a′ and b′ with a and b, respectively, then pointed A′ and B′ on the T2 map raw images according to point A and B to obtain the position of CEP (Fig. 1B). The nutrition of the intervertebral disc is mainly diffused through the center of the cartilage endplate. Biomechanical studies have shown that CEP plays an important role in the redistribution of disc load and stress transmission, while the stress transmitted by the normal disc mainly affects the weakest area in the center of the endplate^26^. In this study, the position of the T2 value area of interest was selected and set in the center of CEP, which is most closely related to discs.

A total of 10 CEP of each subject were enrolled from the lower CEP of L_1_ to the upper CEP of S_1_ and the corresponding ROI was located at A′ and B′. Considering the thinning trend of CEP in adults, the ROI was limited to 4 mm^2^ and the selection of the ROI should avoid the vertebral cortex or disc area. Based on this criterion, the measurement was performed twice for each CEP and took the average value. The schematic diagram of ROI selection is shown in Fig. 1C and the practical measurement is displayed in Fig. 1D.

### *Observation Indicator*

#### *Pfirrmann Grade*

Pfirrmann disc grade is a useful scoring tool for evaluating disc degeneration, which was determined collectively using, for instance, the structure/height of the intervertebral disc, the signal intensity of the nucleus pulposus, and the clarity of boundaries between the nucleus pulposus and the annulus fibrosis. The disc characteristics for each Pm grade are detailed below.

Pm Grade I: Homogeneous structure, bright hyperintense white signal intensity, the nucleus pulposus is clearly demarcated from the annulus fibrosus, normal disc height.

Pm Grade II: Inhomogeneous, normal height, a horizontal gray band could be present, keeping the hyperintense white signal, nucleus and annulus are clearly differentiated.

Pm Grade III: Inhomogeneous, an intermittent gray signal intensity, unclear distinction between nucleus and annulus, normal or slightly declined height.

Pm Grade IV: Inhomogeneous with a hypointense dark gray signal intensity, no more distinction between the nucleus and annulus, slightly or moderately decreased disc height.

Pm Grade V: Inhomogeneous with a hypointense black signal intensity, no more distinction, collapsed disc.

### *Statistical Analysis*

**Figure 2:**
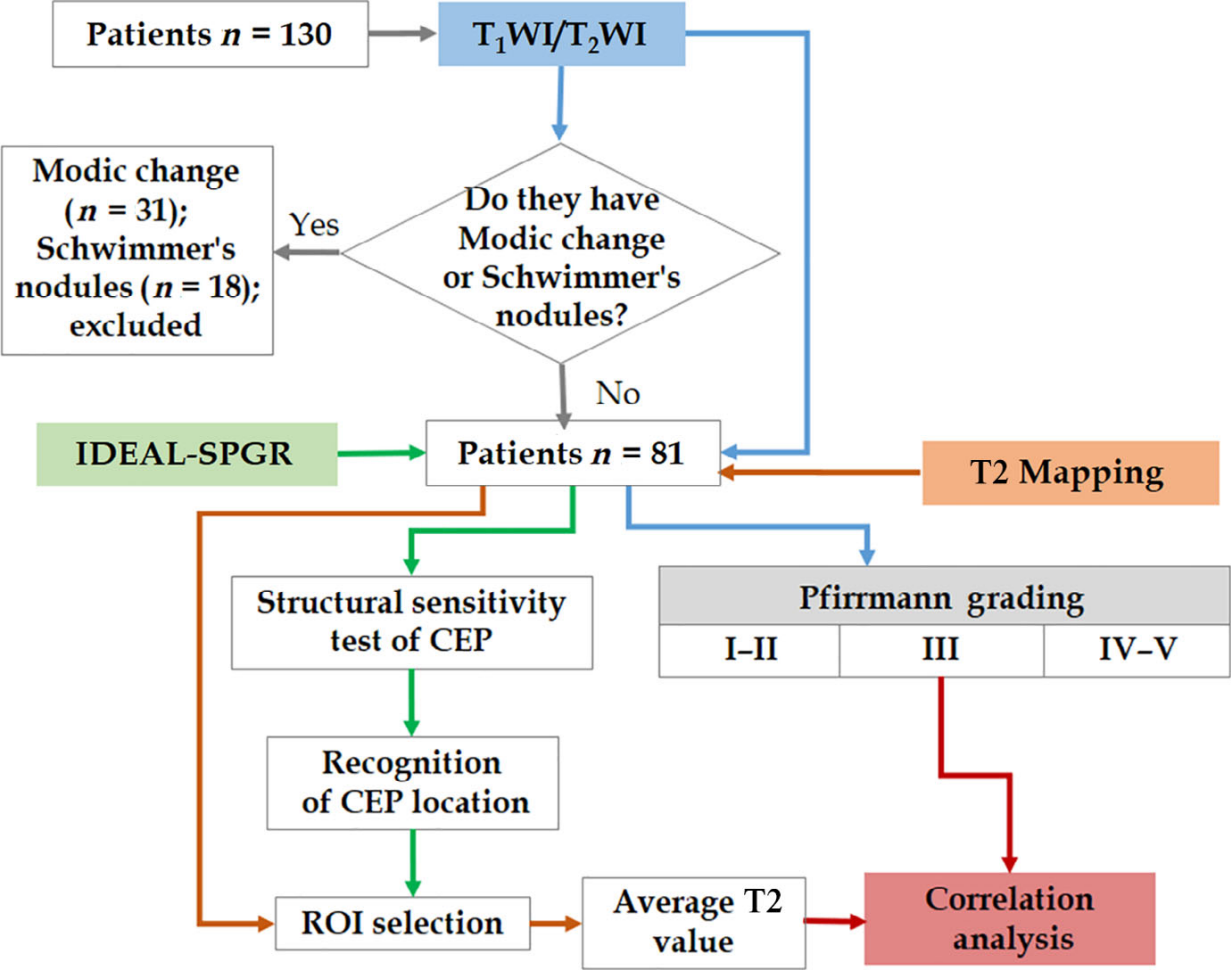
The workflow of the research design.

Statistical analysis was performed on the measured values using SPSS 17.0 statistical software. The T2 values of CEP from L_1_ to S_1_ were represented by mean ± standard deviation and all data conform to the normal distribution. The difference is statistically significant at *P* < 0.05. First, one‐way analysis of variance was used to compare the difference in T2 values of each CEP in G1‐G3 groups. Second, multiple comparisons (least significant difference test) were conducted and the results between groups were compared. Third, the Spearman rank correlation (Spearman’s rho) analysis was used to investigate the correlation between T2 values of CEP and Pm grades, in which *r* > 0.7, 0.5 < *r* ≤ 0.7, and *r* ≤ 0.5 indicate high correlation, moderate correlation, and weak correlation correspondingly. The research design is illustrated in Fig. 2, which is helpful for a comprehensive study the workflow and all methodologies.

## Results

A total of 49 cases observed with lumbar Modic changes or Schmorl nodules were excluded. Modic changes or Schmorl nodules are degenerative changes in the lumbar spine where the completeness and consecutiveness are destroyed, and they could impact the accuracy of T2 value measurement. Of the available 81 cases, 45 were men, with an average age of 45.16 ± 12.20 years, and 36 women, with an average age of 43.33 ± 11.27 years. Ultimately, there were 405 intervertebral discs and 810 CEP enrolled in this statistical study.

### *Pfirrmann Grading for Intervertebral Discs*

**Figure 3:**
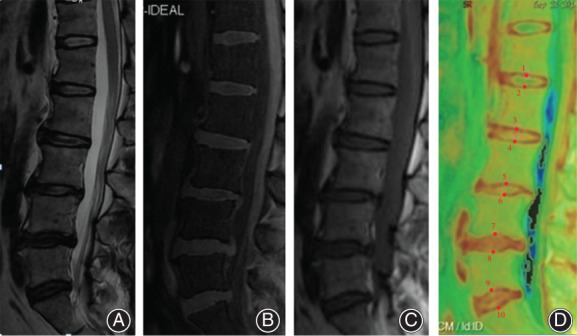
Male, 43 years old. The median sagittal image of T2WI (A) showed that the Pfirrmann (Pm) grades of L_1_–S_1_ intervertebral discs were: L_1–2_ grade II, L_2–3_ grade III, L_3–4_ grade III, L_4–5_ grade IV, and L_5_–S_1_ grade III. The IDEAL‐SPGR image (B) was used for clearly sketching the cartilage endplate (CEP) location and morphology of L_1_–S_1_ intervertebral discs. (C) The T2 value measurement was performed on the T2 mapping original image, (D) marking 10 ROI using numbers in the cephalic and caudal CEP on the T2 mapping pseudo‐color map.

**Figure 4:**
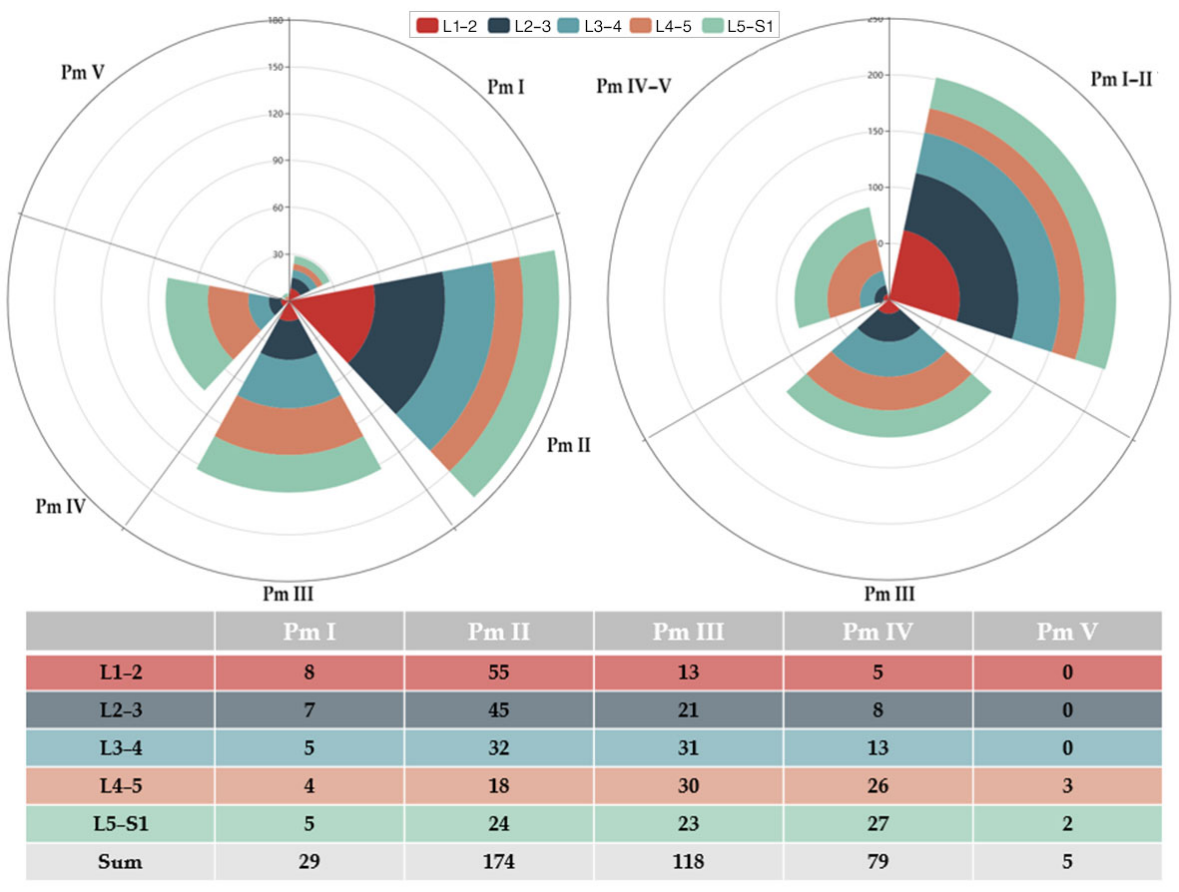
Pfirrmann (Pm) grading results of each intervertebral disc and the distribution in G1–G3 groups.

Figure 3 shows the T2WI sagittal image, the IDEAL‐SPGR scan, the T2 mapping original image, and the T2 mapping pseudo‐color image of 1 subject whose intervertebral discs have different Pm grades. The total 405 intervertebral discs were graded using the Pm grading system according to the disc location and the results are listed in Fig. 4. Moreover, to display the distribution of Pm grading in different groups, we plotted the sum number of G1, G2, and G3 groups simultaneously.

### *Comparison of T2 Values for Cartilage Endplates in G1–G3 Group*

**Figure 5:**
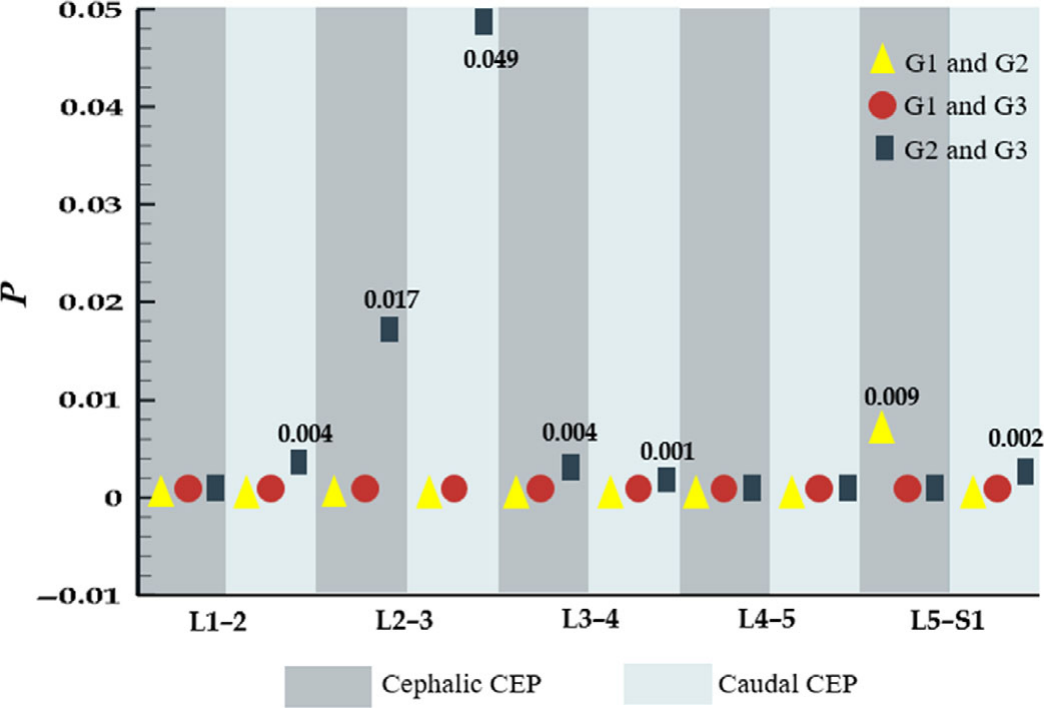
T2 value comparison for each cephalic and caudal cartilage endplate (CEP) between groups.

For each cephalic and caudal CEP, the T2 values in the G1 group were highest, while those in the G2 group were higher than those in the G3 group. The T2 values of each corresponding cartilage endplate among the three groups were all statistically significant (*P* < 0.05, Table 1). Figure 5 compares the T2 values between each two groups and the *P*‐values are represented by different symbols for the three comparisons with different colors (G1 and G2, yellow triangle; G1 and G3, red circle; G2 and G3, rectangle). Meanwhile, nonzero values are denoted with specific values. The results showed that all comparisons had significant differences and G1 and G3 groups had the most significant differences, with all zero *P*‐values. In contrast, the comparison between G2 and G3 groups has the lowest significant difference but the highest *P*‐value (0.049) was still lower than 0.05. For G1 and G2 groups, almost all *P*‐values were zero, except for the cephalic L_5_–S_1_ CEP (*P* = 0.009).

**Table 1:** T2 values of each cephalic and caudal cartilage endplate (CEP) with different Pfirrmann (Pm) levels ($\bar{x}\pm s$, ms)

Location	T2 value			*F* value	*P* value
	Pm I–II	Pm III	Pm IV–V		
L1/2*	61.96 ± 5.89	54.45 ± 3.29	42.47 ± 3.69	25.751	0.000
L1/2†	64.35 ± 5.93	55.28 ± 3.97	44.75 ± 2.12	29.299	0.000
L2/3*	62.96 ± 6.93	55.19 ± 4.02	48.67 ± 4.56	24.415	0.000
L2/3†	65.51 ± 6.49	57.16 ± 5.55	52.05 ± 4.20	24.560	0.000
L3/4*	63.72 ± 5.76	53.96 ± 6.52	48.05 ± 5.00	41.954	0.000
L3/4†	65.46 ± 6.37	55.70 ± 7.50	48.10 ± 3.27	40.757	0.000
L4/5*	66.34 ± 7.68	56.76 ± 9.48	47.80 ± 4.33	38.514	0.000
L4/5†	64.44 ± 4.65	59.30 ± 8.80	47.30 ± 5.78	43.722	0.000
L5/S1*	65.32 ± 5.11	55.33 ± 6.65	48.18 ± 5.37	66.678	0.000
L5/S1†	63.47 ± 4.92	50.32 ± 8.86	44.77 ± 4.69	68.556	0.000

* Cephalic endplate.

† Caudal endplate.

### *Correlation Analysis between T2 Value and Pfirrmann Grading for Each Cartilage Endplates*

**Figure 6:**
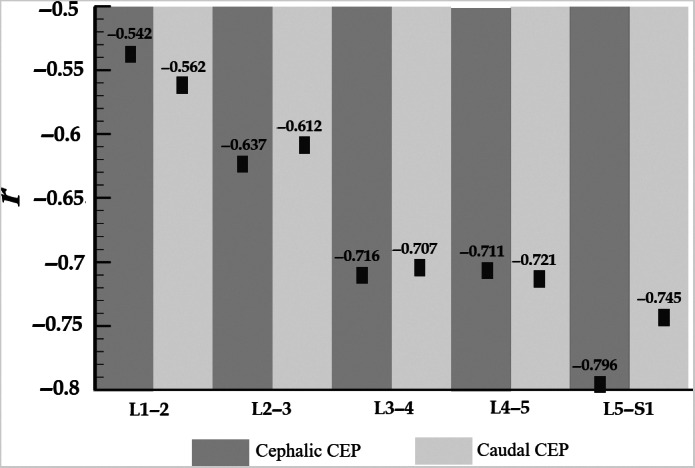
Correlation analysis between T2 value and Pfirrmann (Pm) grading for each cartilage endplate (CEP).

The *P* and *r* values were calculated for each CEP to analyze the correlation between the T2 value and Pm grading. The results are presented in Fig. 6. It should be noted that all *P*‐values equal 0.000 for each CEP. The T2 value of each vertebral endplate was correlated with the Pm grading of the intervertebral disc. The T2 value of higher segment endplates was moderately correlated with the Pm grade of the intervertebral disc. The T2 value of the lower segment endplates was highly correlated with the Pm grade of the intervertebral disc.

## Discussion

Lumbar degenerative osteoarthrosis is a frequently occurring disease in the middle‐aged and elderly population, derived from various reasons but with the early degeneration of intervertebral discs considered the principal factor. The most significant reason might be the obstruction of the nutrition supply between intervertebral discs induced by CEP degeneration. Therefore, in‐depth study of CEP degeneration is valuable for analyzing the mechanism of disc degeneration, which is of great significance in the prevention and early diagnosis of lumbar degenerative osteoarthropathy. A series of factors may cause CEP degeneration, including apoptosis, abnormal stress, genetic abnormalities, inflammatory factors, and aging.

### *Role of Cartilage Endplates in the Degeneration of Intervertebral Discs*

A great many studies have suggested that CEP degeneration plays an important role in the occurrence and development of degenerative disc disease^29^, ^30^. Ariga *et al*.^31^ show that chondrocyte apoptosis occurs in CEP, and the percentage of apoptotic cells increases with age, followed by tearing and disappearance of CEP, where the mechanical stress may be one of the causes resulting in apoptosis. Antoniou *et al*.^32^ found that during the growth phase, the synthesis and catabolism of type II collagen and proteoglycan are relatively strong, while the degeneration of type II collagen is increased, accompanied by type I collagen synthesis, fibrosis in the endplates, and stagnation of metabolites in the degeneration phase^33^. Moreover, matrix synthesis in cartilage endplates reduces with aging and degeneration, when the amount of proteoglycan, water and collagen of the CEP is decreased, and with the collagen fibers arranged disorderly. Consequently, biochemical components of the cartilage endplate undergo qualitative and quantitative changes^34^, ^35^.

These changes inevitably affect the normal physiological function of CEP, further leading to morphological abnormalities such as cartilage calcification, fission, and loss^36^. It has been supported that the histological changes in CEP always precede the histological abnormalities of the nucleus pulposus and there are often significant histological variations in the cartilage endplates before disc degeneration and failure.

Neidlinger‐Wilke *et al*.^37^ indicated that CEP and the nucleus pulposus tissue interact with each other through molecular factors to upregulate matrix‐degrading enzymes and inflammatory factors, thus affecting the pathophysiology of intervertebral disc degeneration. Experimental results showed that abnormal high stress can change the content of proteoglycan in CEP, which hinders the diffusion of sulfur ions into the annulus fibrosus through CEP reducing the content of chondroitin sulfate. In contrast, it promotes self‐calcification, weakening its ability to transmit loads and impedes nutrients from the nucleus pulposus, leading to degeneration of the intervertebral disc^38^, ^39^. Boos *et al*.^2^ found cracks and microfractures in CEP by investigating the histological changes of CEP in cadaveric and intraoperative intervertebral disc samples, which may lead to the decreased blood supply and degeneration of the intervertebral disc.

### *T2 Value as a Biomarker for Cartilage Endplate Imaging*

T2 relaxation time is the attenuation constant of T2 signal intensity in MRI, which is independent of scanner and imaging parameters^16^. It is an inherent property of tissue and reflects the macromolecular environment, including water, protein, collagen, fat, and other solutes. T2 relaxation time is sensitive to water content and the collagen network structure, and is also affected by the dipole interaction caused by the anisotropic movement of water molecules in the collagen matrix^21^. Studies have shown that the T2 value of the nucleus pulposus and fibrous rings of the intervertebral disc is significantly related to the water content and weakly related to the proteoglycan content^16^. The alignment and content of collagen fibers and water content in CEP are the main determinants of the T2 value^20^, ^40^. T2 mapping imaging technology can reflect the tissue macromolecular environment and changes in biochemical components, including water, proteoglycans, and collagen by measuring the T2 relaxation time^36^. This study uses T2 mapping imaging technique to quantitatively measure the T2 value of CEP, and then analyze correlations between CEP degeneration and intervertebral disc degeneration.

### *Relationship between the Degree of Disc Degeneration and T2 Value of Cartilage Endplates*

Structural and compositional changes in CEP are one reason for the intervertebral disc degeneration^41^. In the period of degeneration or aging, the reduction of water, proteoglycan and collagen of CEP and the degree of disarrangement of the collagen fiber network are proportional to the degree of CEP degeneration^42^. Changes in the biochemical composition of CEP can stimulate the formation of cartilage calcification and further reduce the CEP permeability. Subsequently, the substance transport through intervertebral discs is prevented, thus reducing the synthesis of the intervertebral disc matrix and the water content of the nucleus pulposus. This vicious cycle ultimately leads to the degeneration of intervertebral discs and varies in accordance with biomechanical functions^43^. This study showed that the T2 values of the endplates in the Pm grade I–II and III, I–II and IV‐V, and III and IV–V were statistically significant. The T2 value of the cartilage endplate corresponding to degenerative intervertebral discs graded to III and IV–V was significantly lower than that of I–II graded discs. The higher grade of intervertebral disc degeneration and the heavier degeneration degree of CEP result in lower T2 values of adjacent cephalic and caudal cartilage endplates.

It is suggested that when the cartilage endplate degenerates, the cartilage endplate T2 value decreases, the Pm grade increases, and its proteoglycan and water content decrease. Intervertebral disc degeneration has a strong correlation with the T2 value of cartilage endplates, which is consistent with previous studies^7^, indicating that cartilage endplate degeneration is an important factor leading to degeneration of the intervertebral disc.

Muftuler *et al*.^44^ found that the Pm classification was positively correlated with the dynamic MRI enhancement of the CEP and differential enhancement of the cephalic and caudal endplates was also observed. This enhancement increases with increasing Pm classification, and this differential enhancement also depends on the lumbar segment. The subchondral bone that connects the caudal cartilage endplate is thinner than that on the cephalic endplate side. Thinner subchondral bone may be more easily damaged, resulting in more infiltration of contrast media; hence, the enhancement is usually higher in the caudal CEP. In this study, the T2 values of the caudal CEP were higher than for cephalic sides, except L_4–5_ and L_5_–S_1_ intervertebral discs, which needs further investigation.

### *Analysis of T2 Values of Cartilage Endplates in Different Lumbar Segments*

Shirado *et al*.^45^ applied axial load to the lumbar vertebral body, indicating that the maximum bearing pressure of the vertebral body is concentrated in the center of the endplate and the vertebral cancellous bone below it. In addition, when the bearing load exceeds the limit, it will result in the rupture of the central area of the endplate. The overall load gradually increases from L_1–2_ to L_5_–S_1_, so the L_4–5_ and L_5_–S_1_ intervertebral discs bear the highest load. It has been reported that segmental cartilage defects with disc degeneration have segmental differences, which may reflect the characteristics of the maximum load in the lower lumbar region^46^. The present study shows that the lower lumbar disc has a greater degree of degeneration, which is consistent with previous studies.

The upper lumbar segment has more favorable conditions to achieve the balance and regeneration *in vivo*, which may alleviate the deleterious effects of degeneration of CEP in the upper lumbar on the degeneration of the intervertebral disc. Moreover, mechanical factors, like the high load, also affect the CEP permeability to a certain extent, resulting in the structural change of the cartilage endplate. Fields *et al*.^47^ reveal that CEP are affected by high tensile strain and are most prone to the earliest degeneration. Hee *et al*.^48^ suggest that pressurization could lead to CEP degeneration and a decrease in the vascular passage of the bony endplate, which, in turn, results in degeneration of intervertebral discs. Different lumbar segments were subjected to different stresses. This study found that the T2 values of CEP of the L_1–2_ and L_2–3_ segment were moderately correlated with the Pm classification. The T2 values of CEP in the L_4–5_ and L_5_–S_1_ segments were highly correlated with the Pm classification. This might be the result of the higher load on the L_4–5_ and L_5_–S_1_ segments and the subsequent greater stress in the CEP and intervertebral discs. These results imply that different levels of stress can affect the biochemical metabolism of CEP and intervertebral discs.

### *Limitations*

The limitations of this study include, first, that the observation of the morphological variation of CEP was limited by imaging data without the support of surgical or pathological verification. Second, the enrolled cases were derived from patients with chronic low back pain, resulting in an unbalanced Pm distribution (i.e. the concentration in the Pm 2–4 group). In addition, due to the studied subjects being clinical patients, the biochemical changes in CEP degeneration were not studied *in vitro*, which would be necessary in future studies.

### *Conclusion*

T2 values of CEP vary from intervertebral discs with different degeneration levels with a negative correlation, but there is no significant difference between corresponding cephalic and caudal CEP. Measurement of the T2 value of the CEP can be used for the diagnosis of early degeneration of the lumbar intervertebral disc and has important clinical significance for the prevention of disc degeneration.

## References

[1] Vos T , Abajobir AA , Abate KH , *et al* Global, regional, and nationalincidence, prevalence, and years lived with disability for 328 diseasesand injuries for 195 countries, 1990–2016: a systematic analysis forthe global burden of disease study 2016. Lancet, 2017, 390: 1211–1259. 28919117 10.1016/S0140-6736(17)32154-2 PMC5605509

[2] Boos N , Weissbach S , Rohrbach H , Weiler C , Spratt KF , Nerlich AG . Classification of age‐related changes in lumbar intervertebral discs: 2002 volvo award in basic science. Spine (Phila Pa 1976), 2002, 27: 2631–2644. 12461389 10.1097/00007632-200212010-00002

[3] Buchbinder R , Van TM , Öberg B , *et al* Low back pain: a call for action. Lancet, 2018, 391: 2384–2388. 29573871 10.1016/S0140-6736(18)30488-4

[4] Hicks GE , Morone N , Weiner DK . Degenerative lumbar disc and facet disease in older adults: prevalence and clinical correlates. Spine (Phila Pa 1976), 2009, 34: 1301–1306. 19455005 10.1097/BRS.0b013e3181a18263 PMC2867597

[5] Akhtar R , Derby B . Mechanical Properties of Aging Soft Tissues. Cham,Switzerland: Springer International Publishing, 2015; 7–35.

[6] Lotz JC , Fields AJ , Liebenberg EC . The role of the vertebral end plate in low back pain. Global Spine J, 2013, 3: 153–164. 24436866 10.1055/s-0033-1347298 PMC3854605

[7] Magnier C , Boiron O , Wendling‐Mansuy S , Chabrand P , Deplano V . Nutrient distribution and metabolism in the intervertebral disc in the unloaded state: a parametric study. J Biomech, 2009, 42: 100–108. 19110252 10.1016/j.jbiomech.2008.10.034

[8] Xiao L , Ni CL , Shi JD , *et al* Analysis of correlation between vertebral endplate change and lumbar disc degeneration. Med Sci Monit, 2017, 23: 4932–4938. 29032381 10.12659/MSM.904315 PMC5655151

[9] Pfirrmann CW , Metzdorf A , Zanetti M , Hodler J , Boos N . Magnetic resonance classifcation of lumbar intervertebral disc degeneration. Spine (Phila Pa 1976), 2001, 26: 1873–1878. 11568697 10.1097/00007632-200109010-00011

[10] Aigner T , Mckenna L . Molecular pathology and pathobiology of osteoarthritic cartilage. Cell Mol Life Sci, 2002, 59: 5–18. 11846033 10.1007/s00018-002-8400-3 PMC11337501

[11] Eckstein F , Reiser M , Englmeier KH , Putz R . In vivo morphometry and functional analysis of human articular cartilage with quantitative magnetic resonance imagingfrom image to data, from data to theory. Anat Embryol, 2001, 203: 147–173. 11303902 10.1007/s004290000154

[12] Moon SM , Yoder JH , Wright AC , Smith LJ , Vresilovic EJ , Elliott DM . Evaluation of intervertebral disc cartilaginous endplate structure using magnetic resonance imaging. Eur Spine J, 2013, 22: 1820–1828. 23674162 10.1007/s00586-013-2798-1 PMC3731490

[13] Kakitsubata Y , Theodorou DJ , Theodorou SJ , *et al* Cartilaginous endplates of the spine: Mri with anatomic correlation in cadavers. J Comput Assist Tomogr, 2002, 26: 933–940. 12488738 10.1097/00004728-200211000-00013

[14] Siepmann DB , McGovern J , Brittain JH , Reeder SB . High‐resolution 3d cartilage imaging with ideal–spgr at 3T. AJR Am J Roentgenol, 2007, 189: 1510–1515. 18029893 10.2214/AJR.07.2661

[15] Vadapalli R , Mulukutla R , Vadapalli AS , Vedula RR . Quantitative predictive imaging biomarkers of lumbar intervertebral disc degeneration. Asian Spine J, 2019, 13: 1–8. 30966725 10.31616/asj.2018.0166 PMC6680034

[16] Marinelli NL , Haughton VM , Muñoz A , Anderson PA . T_2_ relaxation times of intervertebral disc tissue correlated with water content and proteoglycan content. Spine(Phila Pa 1976), 2009, 34: 520–524. 19247172 10.1097/BRS.0b013e318195dd44

[17] Reeder SB , Pineda AR , Wen ZF , *et al* Iterative decomposition of water and fat with echo asymmetry and least‐squares estimation (ideal): application with fast spin‐echo imaging. Magn Reson Med, 2005, 54: 636–644. 16092103 10.1002/mrm.20624

[18] Disler DG , Peters TL , Muscoreil SJ , *et al* Fat‐suppressed spoiled grass imaging of knee hyaline cartilage. AJR Am J Roentgenol, 1994, 163: 887–892. 8092029 10.2214/ajr.163.4.8092029

[19] Podichetty VK . The aging spine: the role of inflammatory mediators in intervertebral disc degeneration. Cell Mol Biol, 2007, 53: 4–18. 17543240

[20] Gold GE , Burstein D , Dardzinski B , Lang P , Boada F , Mosher T . Mri of articular cartilage in OA: Novel pulse sequences and compositional/functional markers. Osteoarthritis Cartilage, 2006, 14: A76–A86. 16716605 10.1016/j.joca.2006.03.010

[21] Perry J , Haughton V , Anderson P , Wu Y , Fine J , Mistretta C . The value of t2 relaxation times to characterize lumbar intervertebral disks: preliminary results. AJNR Am J Neuroradiol, 2006, 27: 337–342. 16484406 PMC8148766

[22] Wang YX , Zhao F , Griffith JF , *et al* T1rho and T2 relaxation times for lumbar disc degeneration: An in vivo comparative study at 3.0‐tesla mri. Eur Radiol, 2013, 23: 228–234. 22865227 10.1007/s00330-012-2591-2

[23] Watanabe A , Benneker LM , Boesch C , Watanabe T , Obata T , Anderson SE . Classification of intervertebral disk degeneration with axial t2 mapping. AJR Am J Roentgenol, 2007, 189: 936–942. 17885068 10.2214/AJR.07.2142

[24] Takashima H , Takebayashi T , Yoshimoto M , *et al* Correlation between t2 relaxation time and intervertebral disk degeneration. Skeletal Radiol, 2012, 41: 163–167. 21424906 10.1007/s00256-011-1144-0

[25] Haughton V . Imaging intervertebral disc degeneration. J Bone Joint Surg Am, 2006, 88: 15–20. 16595437 10.2106/JBJS.F.00010

[26] Grant JP , Oxland TR , Dvorak MF . Mapping the structural properties of the lumbosacral vertebral endplates. Spine (Phila Pa 1976), 2001, 26: 889–896. 11317111 10.1097/00007632-200104150-00012

[27] Shirado O , Kaneda K , Tadano S , Ishikawa H , Mcafee PC , Warden KE . Influence of disc degeneration on mechanism of thoraco lumbar burst fractures. Spine (Phila Pa 1976), 1992, 17: 286–292. 1566166 10.1097/00007632-199203000-00008

[28] Hughes SPF , Freemont AJ , Hukins DWL , Mcgregor AH , Roberts S . The pathogenesis of degeneration of the intervertebral disc and emerging therapies in the management of back pain. J Bone Joint Surg Br, 2012, 94: 1298–1304. 23015552 10.1302/0301-620X.94B10.28986

[29] Rajasekaran S , Venkatadass K , BabuNJ GK , Shetty AP . Pharmacological enhancement of disc diffusion and differentiation of healthy, ageing and degenerated discs: results from in vivo serial post‐contrast MRI studies in 365 human lumbar discs. Eur Spine J, 2008, 17: 626–643. 18357472 10.1007/s00586-008-0645-6 PMC2367412

[30] Boyd LM , Carter AJ . Injectable biomaterials and vertebral endplate treatment for repair and regeneration of the intervertebral disc. Eur Spine J, 2006, 15: 414–421. 10.1007/s00586-006-0172-2 PMC2335387 16868785

[31] Ariga K , Miyamoto S , Nakase T , *et al* The relationship between apoptosis of endplate chondrocytes and aging and degeneration of the intervertebral disc. Spine (Phila Pa 1976), 2001, 26: 2414–2420. 11707702 10.1097/00007632-200111150-00004

[32] Antoniou J , Goudsouzian NM , Heathfield TF , *et al* The human lumbar endplate: Evidence of changes in biosynthesis and denaturation of the extracellular matrix with growth,maturation, aging, and degeneration. Spine (Phila Pa 1976), 1996, 21: 1153–1161. 8727189 10.1097/00007632-199605150-00006

[33] Van der Werf M , Lezuo P , Maissen O , Van Donkelaar C , Ito K . Inhibition of vertebral endplate perfusion results in decreased intervertebral disc intranuclear diffusive transport. J Anat, 2007, 211: 769–774. 17953653 10.1111/j.1469-7580.2007.00816.x PMC2375848

[34] Bernick S , Cailliet R . Vertebral end‐plate changes with aging of human vertebrae. Spine (Phila Pa 1976), 1982, 7: 97–102. 7089697 10.1097/00007632-198203000-00002

[35] Bishop PB , Pearce RH . The proteoglycans of the cartilaginous end‐plate of the human intervertebral disc change after maturity. J Orthop Res, 1993, 11: 324–331. 8326438 10.1002/jor.1100110303

[36] Berg‐Johansen B , Han M , Fields AJ , *et al* Cartilage endplate thickness variation measured by ultrashort echo‐time mri is associated with adjacent disc degeneration. Spine (Phila Pa 1976), 2018, 43: 592–600. 10.1097/BRS.0000000000002432 PMC5882595 28984733

[37] Neidlinger‐Wilke C , Boldt A , Brochhausen C , *et al* Molecular interactions between human cartilaginous endplates and nucleus pulposus cells: a preliminary investigation. Spine (Phila Pa 1976), 2014, 39: 1355–1364. 24831500 10.1097/BRS.0000000000000372

[38] Siemionow K , An H , Masuda K , Andersson G , Cs‐Szabo G . The effects of age, sex, ethnicity, and spinal level on the rate of intervertebral disc degeneration: a review of 1712 intervertebral discs. Spine (Phila Pa 1976), 2011, 36: 1333–1339. 21217432 10.1097/BRS.0b013e3181f2a177 PMC3117081

[39] Benneker LM , Heini PF , Alini M , Anderson SE , Ito K . 2004 young investigator award winner: vertebral endplate marrow contact channel occlusions and intervertebral disc degeneration. Spine (Phila Pa 1976), 2005, 30: 167–173. 15644751 10.1097/01.brs.0000150833.93248.09

[40] Nguyen JC , Liu F , Blankenbaker DG , Woo KM , Kijowski R . Juvenile osteochondritis dissecans: cartilage t2 mapping of stable medial femoral condyle lesions. Radiology, 2018, 288: 536–543. 29762089 10.1148/radiol.2018171995 PMC6067819

[41] Roberts S , Menage J , Urban JP . Biochemical and structural properties of the cartilage endplate and its relation to the intervertebral disc. Spine (Phila Pa 1976), 1989, 14: 166–174. 2922637 10.1097/00007632-198902000-00005

[42] Cinotti G , Della RC , Romeo S , Vittur F , Toffanin R , Trasimeni G . Degenerative changes of porcine intervertebral disc induced by vertebral endplate injuries. Spine (Phila Pa 1976), 2005, 30: 174–180. 15644752 10.1097/01.brs.0000150530.48957.76

[43] Hebelka H , Miron A , Kasperska I , Brisby H , Lagerstrand K . Axial loading during mri induces significant t2 value changes in vertebral endplates‐a feasibility study on patients with low back pain. J Orthop Surg Res, 2018, 13: 18. 29378613 10.1186/s13018-018-0727-z PMC5789539

[44] Muftuler LT , Jarman JP , Hon JY , Gardner VO , Maiman DJ , Arpinar VE . Association between intervertebral disc degeneration and endplate perfusion studied by DCE‐MRI. Eur Spine J, 2015, 24: 679–685. 25421547 10.1007/s00586-014-3690-3

[45] Shirado O , Kaneda K , Tadano S , Ishikawa H , McAFEE PC , Warden KE . Influence of disc degeneration on mechanism of thoracolumbar burst fractures. Spine (Phila Pa 1976), 1992, 17: 286–292. 1566166 10.1097/00007632-199203000-00008

[46] Law T , Anthony MP , Chan Q , *et al* Ultrashort time‐to‐echo mri of the cartilaginous endplate: technique and association with intervertebral disc degeneration. J Med Imaging Radiat Oncol, 2013, 57: 427–434. 23870338 10.1111/1754-9485.12041

[47] Fields AJ , Lee GL , Keaveny TM . Mechanisms of initial endplate failure in the human vertebral body. J Biomech, 2010, 43: 3126–3131. 20817162 10.1016/j.jbiomech.2010.08.002 PMC2991488

[48] Hee HT , Chuah YJ , Tan BH , Setiobudi T , Wong HK . Vascularization and morphological changes of the endplate after axial compression and distraction of the intervertebral disc. Spine (Phila Pa 1976), 2011, 36: 505–511. 20975621 10.1097/BRS.0b013e3181d32410

